# A riddle on norA, norB, and norC

**DOI:** 10.1128/spectrum.01834-24

**Published:** 2025-01-14

**Authors:** Theresa Sievers, Christina Susanne Hölzel

**Affiliations:** 1 Faculty of Agricultural and Nutritional Sciences, Institute for Animal Breeding and Husbandry, Kiel University, Kiel, Germany; Innovations Therapeutiques et Resistances, Toulouse, France

**Keywords:** antimicrobial resistance, ARG, AMR, *nor*A, fluoroquinolone, PCR, quality control, *Staphylococcus aureus*

## LETTER

We contact you concerning a publication of Zhang et al. (1). In that publication, the authors report on the detection of antibiotic resistance genes (ARGs) in *Staphylococcus aureus* from bovine mastitis. In particular, the authors ask us a riddle on *nor*A/*nor*B/*nor*C that we cannot solve without their help.

According to CARD (https://card.mcmaster.ca/ontology/36530, last accessed 23 July 2024), NorA is a multidrug efflux pump in *S. aureus* that confers resistance to fluoroquinolones. It does so particularly in case of mutation or upregulation, while the gene itself is chromosomally encoded, conferring only low-level resistance (2). The CARD reference sequences for staphylococcal *nor*A and *nor*C are present in the reference representative *S. aureus* genome (*nor*A: NC_007795.1: 687425–688591, 100% query cover [QC] and 99.9% identity [ID]; *nor*C: NC_007795.1: 62384–63772, 100% QC, 96% ID). For staphylococcal *nor*B, no valid GenBank entry is provided, but the listerial CARD reference protein aligns with 97% QC and 57% ID with the multispecies NorB efflux protein WP_000414682.1 from *Staphylococcu*s, which is encoded in the reference representative genome of *S. aureus* NC_007795.1 (1405099–1406487, 100% QC, 99% ID, if not filtered for low-complexity regions; otherwise 80%). As expected for intrinsic, core genome features, all three variants are also encoded in ≥5,000 other *S. aureus* genomes (5000 is the limit of the Basic Local Alignment Serach Tool [BLAST] of the National Center for Biotechnology Information [NCBI]).

So, given the fixation of *nor* genes in the *S. aureus* core genome, we were wondering why the authors did not find these chromosomal genes in roughly half of the isolates. To solve that, we wanted to check the primers in a primer BLAST search. However, when we did so, we noted that the primer reference for *nor*A/*nor*B/*nor*C does not include *nor*A/*nor*B/*nor*C primers.

There is a deeper layer implied in the unsuccessful amplification of chromosomal genes: if the authors searched for staphylococcal *nor*A/*nor*B/*nor*C but did not find it in nearly half of the isolates, have those isolates been *S. aureus* at all? Initial species identification seems rather weak, combining a chromogenic medium with 16S-amplicon sequencing of an unspecified region. Anyhow, the absence of *nor*A/*nor*B/*nor*C amplicons might have also been related to a variability of primer-binding regions, since different gene variants exist in *S. aureus*, such as *nor*AI (D90119.1), *nor*AII (norA23, AB019536.1), and *nor*AIII (M97169.1) (2), with 89%–93% ID to each other in our NCBI BLAST search. Of these, *nor*AI is the most common and also present in the reference representative genome of *S. aureus* NC_007795.1

Finally, getting an answer on primers would only solve half of the riddle, since the more prominent question is why did the authors search for a chromosomal ARG at all? Obviously, they did not assess any features that might affect its functionality, such as mutations or overexpression. While intrinsic ARGs are an unavoidable by-product in whole-genome sequencing analysis, we cannot see any reason for amplifying them by PCR. Thus, we would suggest to be more critical in the choice of target ARGs, simply for reasons of cost efficiency and scientific impact.
